# Malignant solitary fibrous tumor occurring in the mediastinal pleura showing NAB2ex4‐STAT6ex2 fusion and negative STAT6 immunohistochemistry: A case report

**DOI:** 10.1111/1759-7714.13395

**Published:** 2020-03-20

**Authors:** Peng Zhang, Kai Xiong, Peng Lv, Hui Zhang, Yuanguo Wang, Zhaoyu Yang, Ziyou Tao, Peng Zhang, Wenjing Song

**Affiliations:** ^1^ Department of Cardiothoracic Surgery Tianjin Medical University General Hospital Tianjin China

**Keywords:** Mediastinal pleura, solitary fibrous tumor, STAT6, surgery

## Abstract

Solitary fibrous tumor (SFT) is a rare clinical tumor, defined as a mesenchymal tumor of fibroblastic origin. A classic SFT is benign in most cases, but its clinical behavior is unpredictable. Lately, molecular analyses has discovered that almost all SFTs harbor an NAB2‐STAT6 fusion gene, which is considered specific to this tumor type. Recent studies have suggested that nuclear STAT6 immunoreactivity is a highly sensitive and specific marker of SFTs and can be helpful when diagnosis is inconclusive by conventional methods. We herein report the case of a rare malignant solitary fibrous tumor occurring in the mediastinal pleura. An 82‐year‐old Chinese man with intermittent breathlessness was referred to our hospital. Chest CT showed a significantly enhanced irregular huge soft tissue mass in the anterior mediastinal area. After radical resection, the immunohistochemistry staining results of the sample showed that STAT6 was negative. The final diagnosis was confirmed by qualitative endpoint reverse transcriptase‐polymerase chain reaction technique, showing positive NAB2ex4‐STAT6ex2 fusion.

## Introduction

Solitary fibrous tumor (SFT) is a soft tissue neoplasm that was initially described in the pleura. It is a rare clinical tumor accounting for 4% of chest tumors. This type of tumor originates from mesothelial cells, can occur in any part of the body, but location in the visceral pleura is common, accounting for 80% of all pleura SFTs.^1^ Although most SFTs with classical morphologic features behave in an indolent manner, and SFTs with obvious malignant histological features tend to be aggressive neoplasms that behave as high‐grade sarcomas, the behavior of SFT is unpredictable. Only a few SFTs with typical pathological manifestations are aggressive and require long‐term clinical follow‐up.^2^ However, anterior mediastinal pleura SFTs are rare. Only nine case reports of the anterior mediastinal SFT were retrieved on PUBMED. Our study focuses on the clinical presentation, histopathological and immunohistochemical diagnosis, molecular analyses, and review of the available literature regarding this rare tumor.

## Case report

**Figure 1:**
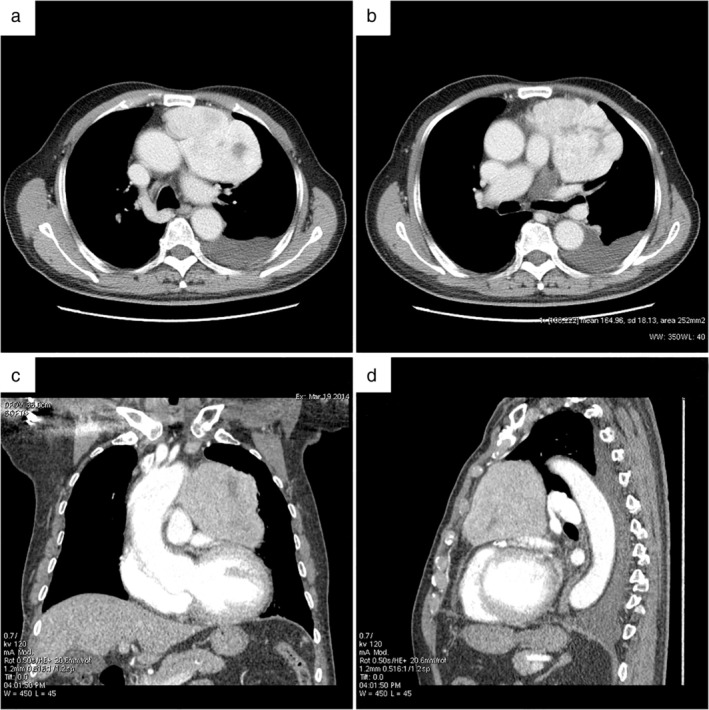
Chest computed tomography (CT) scan showed a significantly enhanced irregular huge soft tissue mass in the anterior mediastinal area (**a–d**).

**Figure 2:**
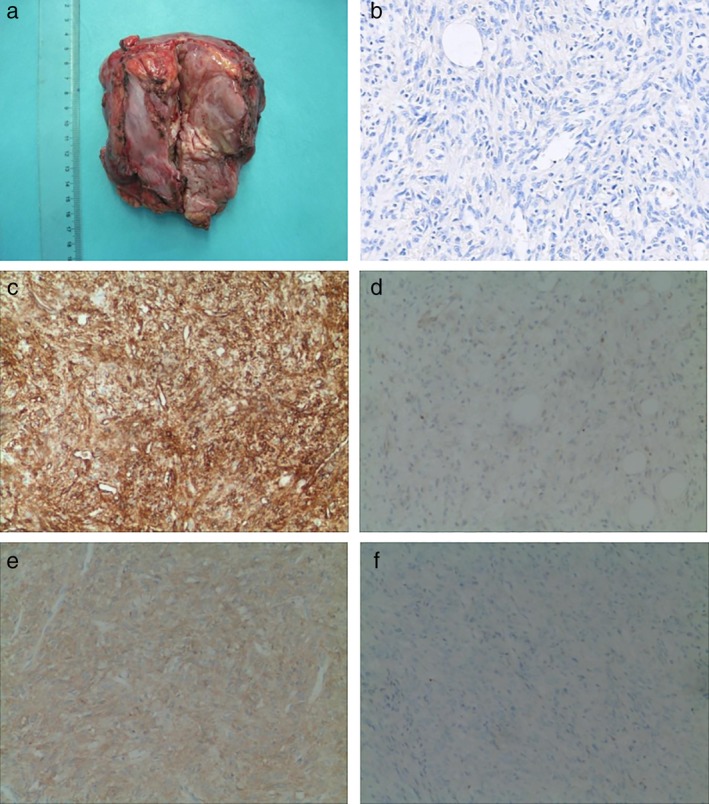
(**a**) Macroscopic findings of resected tumor: The tumor measured approximately 10 cm × 9 cm × 6 cm, and the capsule was incomplete with a rich blood supply. (**b**–**e**) Immunohistochemical staining: (**b**) Tumor cells were negative for STAT6 (100X); (**c**) tumor cells showing strong positivity for CD34 (100X); (**d**) tumor cells showing partial positivity for Bcl‐2 (100X); (**e**) tumor cells showing diffuse positivity for CD99 (100X), and (**f**) tumor cells negative for s‐100 (100X).

An 82‐year‐old male presented to our facility on 27 May 2014 with a history of intermittent breathlessness with exacerbation of symptoms after exercise and improvement after rest. The patient had undergone coronary stent implantation seven years previously for extensive anterior myocardial infarction, with accompanying chronic cardiac insufficiency. A chest computed tomography (CT) scan was carried out at the clinic which showed a significantly enhanced irregular huge soft tissue mass in the anterior mediastinal area, considered likely to be a malignant lesion (Fig 1). A CT‐guided transthoracic fine‐needle aspiration was performed at the left second intercostal space and the biopsy specimen showed pathological findings consistent with a spindle cell tumor with mitotic figures (MF) up to 14/10 high power fields (HPF). Immunohistochemistry results demonstrated that CD34 and vimentin were strong positive, Bcl‐2 was partially positive and CD99 was diffuse positive, while STAT6, melanocyte, cytokeratin, epithelial cell membrane antigen, S‐100, and calretinin were all negative (Fig 2). A diagnosis of solitary fibrous tumor (SFT) was suspected as a result of the negative immunohistochemistry staining of STAT6. Following surgery via a sternal midline incision, the tumor was found to be located at the left side of the anterior mediastinum, pulmonary artery and pericardium. It invaded the left mediastinal pleura and pericardium and was closely related to the left phrenic nerve. There was no invasion of the lungs, large blood vessels and no metastasis to the chest. The tumor was completely removed. After a difficult perioperative period, the patient improved and was discharged. Postoperative pathological results were consistent with those before surgery. The aggressive growth of this tumor indicated its potentially malignant biological behavior. Because immunohistochemistry staining of STAT6 was negative, frozen tissue sections were tested for eight fusion variants of NAB2‐STAT6, using qualitative endpoint reverse transcriptase‐polymerase chain reaction technique. RNA extraction was performed using the RecoverAll Total Nucleic Acid Isolation kit (Code No.: 9108Q; RNAiso Plus, Takara). PCR primers were designed according to the previous reports. The tumor showed NAB2ex4‐STAT6ex2 fusion transcript (Fig 4). Finally, a diagnosis of malignant solitary fibrous tumor occurring in the mediastinal pleura was confirmed.

**Figure 3:**
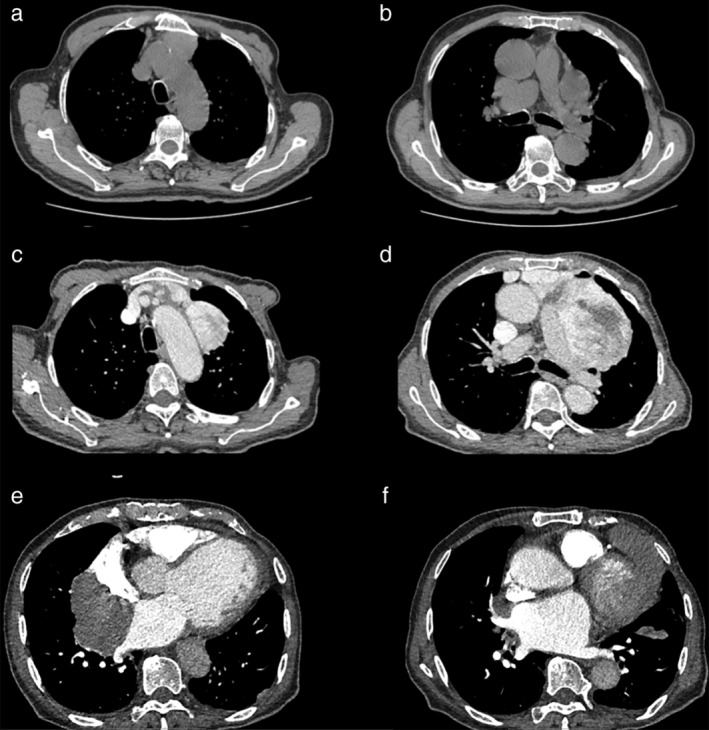
Chest computed tomography (CT) scan showed multiple recurrences. (**a,b**) On chest CT 22 July 2015, two soft tissue density masses were seen in the anterior mediastinal and left pulmonary artery areas. (**c,d**) On 1 April 2016 chest CT showed that two masses were significantly enlarged and locally fused. (**e,f**) On 1 April 2016, chest CT showed multiple new masses of different sizes had appeared in the right lower hilum and both sides of the heart. Part of the mass protruded into the lungs. Fusiform soft tissue was seen in the left side interlobar fissure pleura.

On chest CT on 22 July 2015, two soft tissue density masses were seen in front of the mediastinal thymus and left pulmonary artery area. Because of significant cardiac dysfunction, a second operation was not performed. Chest CT on the 1 April 2016 showed that the two masses were significantly enlarged and locally fused. The boundary between the masses, the left atrial appendage, and the right atrium wall was unclear. Multiple new masses of different sizes were apparent in the right lower hilum and both sides of the heart. Part of the mass was seen to be protruding into the lungs. Fusiform soft tissue was seen in the left side interlobar fissure pleura (Fig 3), and recurrence and metastatic lesions were more significant than previously. Due to the severe heart failure in this patient, the antitumor treatment was not performed and the patient died in March 2017.

## Discussion

SFT is a rare mesenchymal tumor. The 2013 WHO classification criteria for bone and soft tissue tumors classifies SFT in the fibroblast/myofibroblastic tumor group. The biological behavior of SFT is mostly benign, and a few tumors are potentially malignant. Approximately 10% to 15% of SFTs are considered to show invasive behavior. Therefore, SFT is defined as an intermediate tumor with local recurrence, which rarely metastasizes.^3^, ^4^

Mohajeri *et al*. and Tai *et al*. performed molecular genetic analysis on 44 cases and 88 cases of SFT, respectively, and found that the 3′ end of the NAB2 gene located on chromosome 12 was inverted with the 5′ end of the STAT6 gene, resulting in NAB2‐STAT6 gene fusion. It confirmed the existence of high frequency gene fusion mutations in SFT, suggesting that the fusion gene may be an important molecular event leading to the development of SFT.^5^ Gene sequencing, reverse transcriptase polymerase chain reaction and immunohistochemistry showed that the NAB2⁃STAT6 fusion gene can lead to strong nuclear signal expression of STAT6, suggesting that immunohistochemistry and gene sequencing results are highly consistent. Therefore, STAT6 immunohistochemical staining can reliably replace the detection of the NAB2⁃STAT6 fusion gene under the premise of ensuring the accuracy of the test results. Schweizer *et al*. and Yoshida *et al*. used immunohistochemistry to detect the expression of STAT6 in SFT. The positive rates were 96.8% (60/62) and 100% (49/49), indicating that STAT6 has a higher sensitivity for SFT detection. STAT6 test results are of great value for diagnosis, especially for CD34‐negative SFT cases.^6^, ^7^, ^8^ However, we should know that STAT6 immunohistochemistry staining could not replace the detection of the NAB2‐STAT6 fusion gene completely.

**Figure 4:**
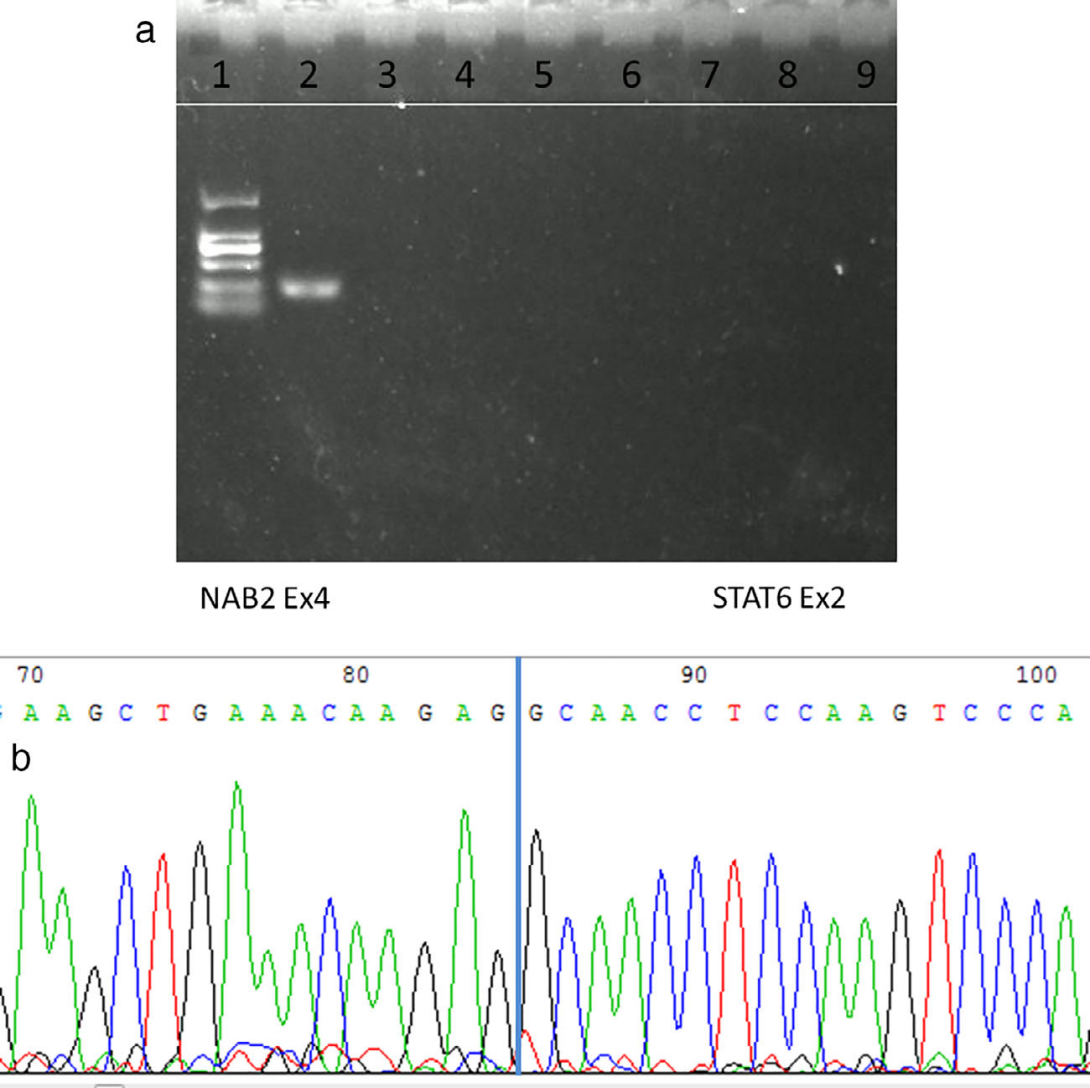
(**a**) Conventional reverse transcriptase‐polymerase chain reaction for NAB2‐STAT6 fusion transcripts analyzed on 12% PAGE. Lane 1: 100–2000 bp molecular weight marker. Lane 2: Case positive for NAB2‐STAT6 exon3‐exon2 (exon4‐exon 2 fusion type; 233 bp). (**b**) The sequencing results of the recovered PCR products showed NAB2‐STAT6 fusion (exon 4‐exon 2 fusion type).

In the case reported here, immunohistochemistry results demonstrated that CD34 and vimentin were strong positive, while STAT6 was negative. In order to confirm the diagnosis, frozen tissue sections were tested for eight fusion variants of NAB2‐STAT6, using qualitative endpoint reverse transcriptase‐polymerase chain reaction technique. RNA extraction was performed using RecoverAll Total Nucleic Ncid Isolation kit. PCR primers were designed according to the previous reports. The tumor showed NAB2ex4‐STAT6ex2 fusion transcript (Fig 4). Finally, we confirmed that it was a malignant solitary fibrous tumor occurring in the mediastinal pleura.

Few SFT cases have been reported negative for STAT6 detection. The current classification of SFT includes usual, malignant and the recently described dedifferentiated variants. The rare dedifferentiated variant is characterized by the appearance of a high‐grade component mimicking a pleomorphic/spindle cell sarcoma, small‐cell sarcoma and other entities that have no morphological resemblance to solitary fibrous tumors. This unusual component may be present at the same time as a primary malignant or usual solitary fibrous tumor, or appear often many years later during subsequent recurrences. Dagrada *et al*. found that immunohistochemistry and western blot analysis indicated that chimeric protein expression was atypical or absent in nine out of 11 dedifferentiated tumors. This suggests that the loss of STAT6 is related to the dedifferentiation of the SFT.^9^, ^10^

In conclusion, we report a rare malignant solitary fibrous tumor occurring in the mediastinal pleura showing positive NAB2ex4‐STAT6ex2 fusion and negative STAT6 immunohistochemistry staining. Therefore, this suggests that the absence of nuclear STAT6 expression by immunohistochemistry staining in the anterior mediastinal mass may not exclude the possibility of SFT.

## Disclosure

No authors report any conflict of interest.

## References

[1] You YH , Liu RT , Zhang Y . A large solitary fibrous tumour of the pleura: A case report and review of the literature. J Int Med Res 2018; 46: 1672–7. 29376453 10.1177/0300060517750534 PMC6091813

[2] Thway K , Ng W , Noujaim J , Jones RL , Fisher C . The current status of solitary fibrous tumor: Diagnostic features, variants, and genetics. Int J Surg Pathol 2016; 24: 281–92. 26811389 10.1177/1066896915627485

[3] Hui M , Xu Y , Zhang N , He XD , Qu Q . [Clinical characteristics of abdominal solitary fibrous tumor: An analysis of 18 cases]. Zhonghua Yi Xue Za Zhi 2018; 98: 1439–42. 29804409 10.3760/cma.j.issn.0376-2491.2018.18.014

[4] Ronchi A , Cozzolino I , Zito MF *et al* Extrapleural solitary fibrous tumor: A distinct entity from pleural solitary fibrous tumor. An update on clinical, molecular and diagnostic features. Ann Diagn Pathol 2018; 34: 142–50. 29660566 10.1016/j.anndiagpath.2018.01.004

[5] Huang SC , Huang HY . Solitary fibrous tumor: An evolving and unifying entity with unsettled issues. Histol Histopathol 2019; 34: 313–34. 30431144 10.14670/HH-18-064

[6] Rekhi B , Shetty O , Tripathi P *et al* Molecular characterization of a series of solitary fibrous tumors, including immunohistochemical expression of STAT6 and NATB2‐STAT6 fusion transcripts, using Reverse Transcriptase (RT)‐Polymerase chain reaction (PCR) technique: An Indian experience. Pathol Res Pract 2017; 213: 1404–11. 28869107 10.1016/j.prp.2017.08.011

[7] Schweizer L , Koelsche C , Sahm F *et al* Meningeal hemangiopericytoma and solitary fibrous tumors carry the NAB2‐STAT6 fusion and can be diagnosed by nuclear expression of STAT6 protein. Acta Neuropathol 2013; 125: 651–8. 23575898 10.1007/s00401-013-1117-6

[8] Yoshida A , Tsuta K , Ohno M *et al* STAT6 immunohistochemistry is helpful in the diagnosis of solitary fibrous tumors. Am J Surg Pathol 2014; 38: 552–9. 24625420 10.1097/PAS.0000000000000137

[9] Schneider N , Hallin M , Thway K . STAT6 loss in dedifferentiated solitary fibrous tumor. Int J Surg Pathol 2017; 25: 58–60. 27189111 10.1177/1066896916650257

[10] Dagrada GP , Spagnuolo RD , Mauro V *et al* Solitary fibrous tumors: Loss of chimeric protein expression and genomic instability mark dedifferentiation. Mod Pathol 2015; 28: 1074–83. 26022454 10.1038/modpathol.2015.70

